# Holohemispheric Intracranial Subdural Empyema as a Complication of Orbital Cellulitis

**DOI:** 10.7759/cureus.59065

**Published:** 2024-04-26

**Authors:** Hadi Abou-El-Hassan, Yosof Katiby, Elias A Giraldo

**Affiliations:** 1 Neurology, Arrowhead Regional Medical Center, Colton, USA; 2 Neurology, California University of Science and Medicine, Colton, USA

**Keywords:** infection, empyema, orbital cellulitis, neurosurgery, subdural empyema

## Abstract

Intracranial subdural empyema is a loculated collection of pus in the subdural space between the dura mater and the arachnoid that can be life-threatening. Here, we present a case of a 22-year-old man hospitalized for management of sepsis due to right orbital cellulitis who experienced sudden-onset right-sided hemiplegia and was found to have a holohemispheric intracranial subdural empyema requiring emergent neurosurgical intervention. Subdural empyemas are commonly caused by maxillofacial infections, including orbital infections. We demonstrate that orbital cellulitis may cause an intracranial subdural empyema that can present with sudden-onset neurological deficits warranting prompt neurosurgical intervention.

## Introduction

An intracranial subdural empyema refers to a confined buildup of infected material, specifically pus, found in the space between the dura mater and the arachnoid membrane within the skull [1]. Although often caused by maxillofacial infections such as sinusitis, mastoiditis, and otitis, the etiology of subdural empyema also includes traumatic skull fractures as well as post-surgical complications [1]. Subdural empyema can be life-threatening, and a swift diagnosis and treatment are essential to mitigate the high rates of complications and mortality associated with this condition [2].

Intracranial subdural empyema typically impacts children and young adults, with males being more frequently affected compared to females [2, 3]. While subdural empyemas can occasionally occur in the spinal canal, such cases are seldom reported. Furthermore, intracranial subdural empyemas are notably less prevalent than brain abscesses [2]. Here, we present a case of a 22-year-old man hospitalized for management of sepsis due to right orbital cellulitis who experienced sudden-onset right-sided hemiplegia and was found to have a holohemispheric intracranial subdural empyema requiring emergent neurosurgical intervention.

## Case presentation

**Figure 1:**
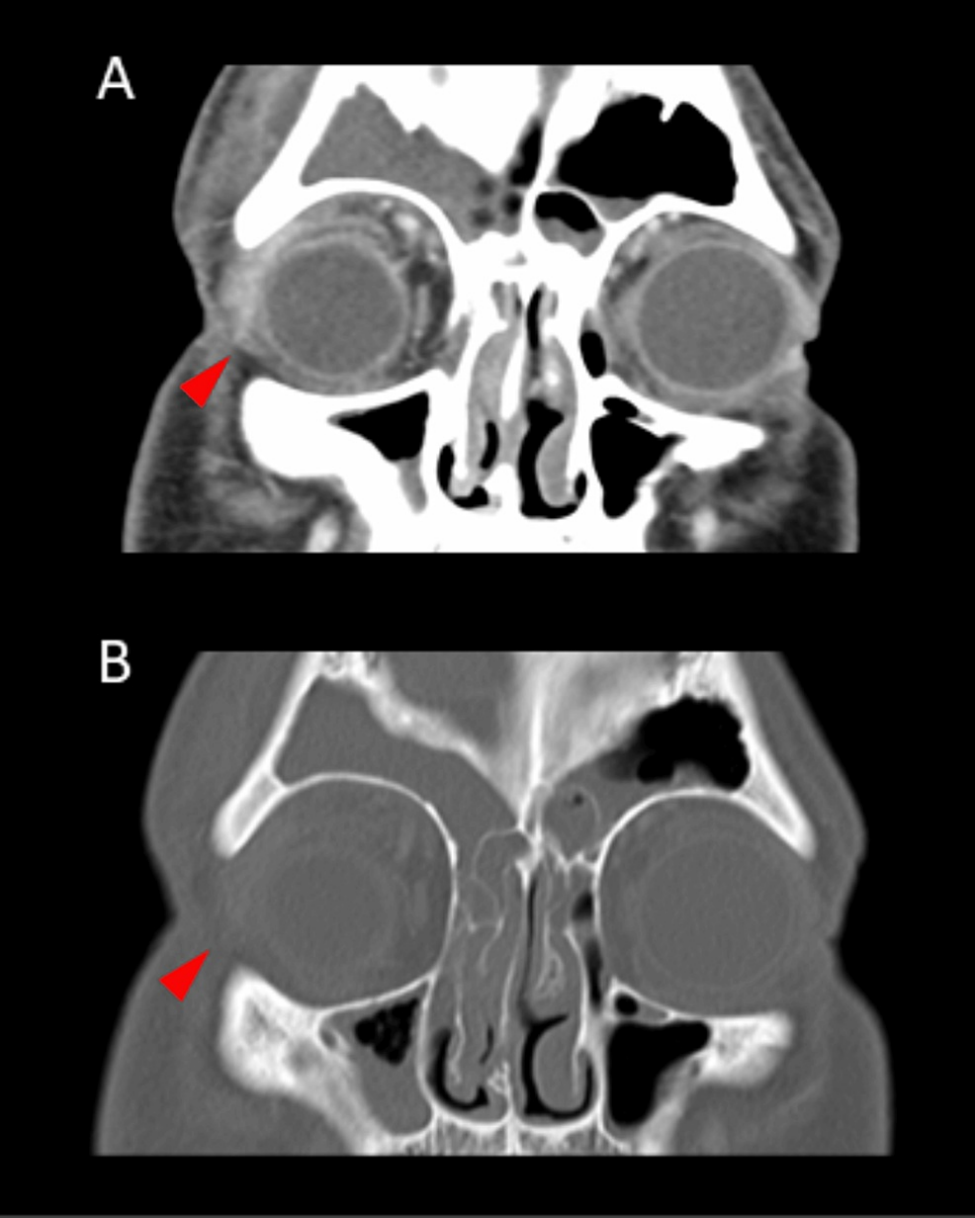
Imaging of the orbital cellulitis A CT scan of the orbits with contrast (A, B) shows right orbital soft tissue swelling (red arrowheads) and compression of the right eyeball.

A 22-year-old right-handed Hispanic man presented to the emergency room with a five-day history of fever and right-sided eye pain and redness. A CT scan of the orbits revealed right orbital cellulitis (Figures 1A, 1B), and laboratory workup revealed leukocytosis with neutrophilic predominance. The patient was then started on empiric antibiotics and was admitted to the hospital for the management of sepsis due to right orbital cellulitis.

**Figure 2:**
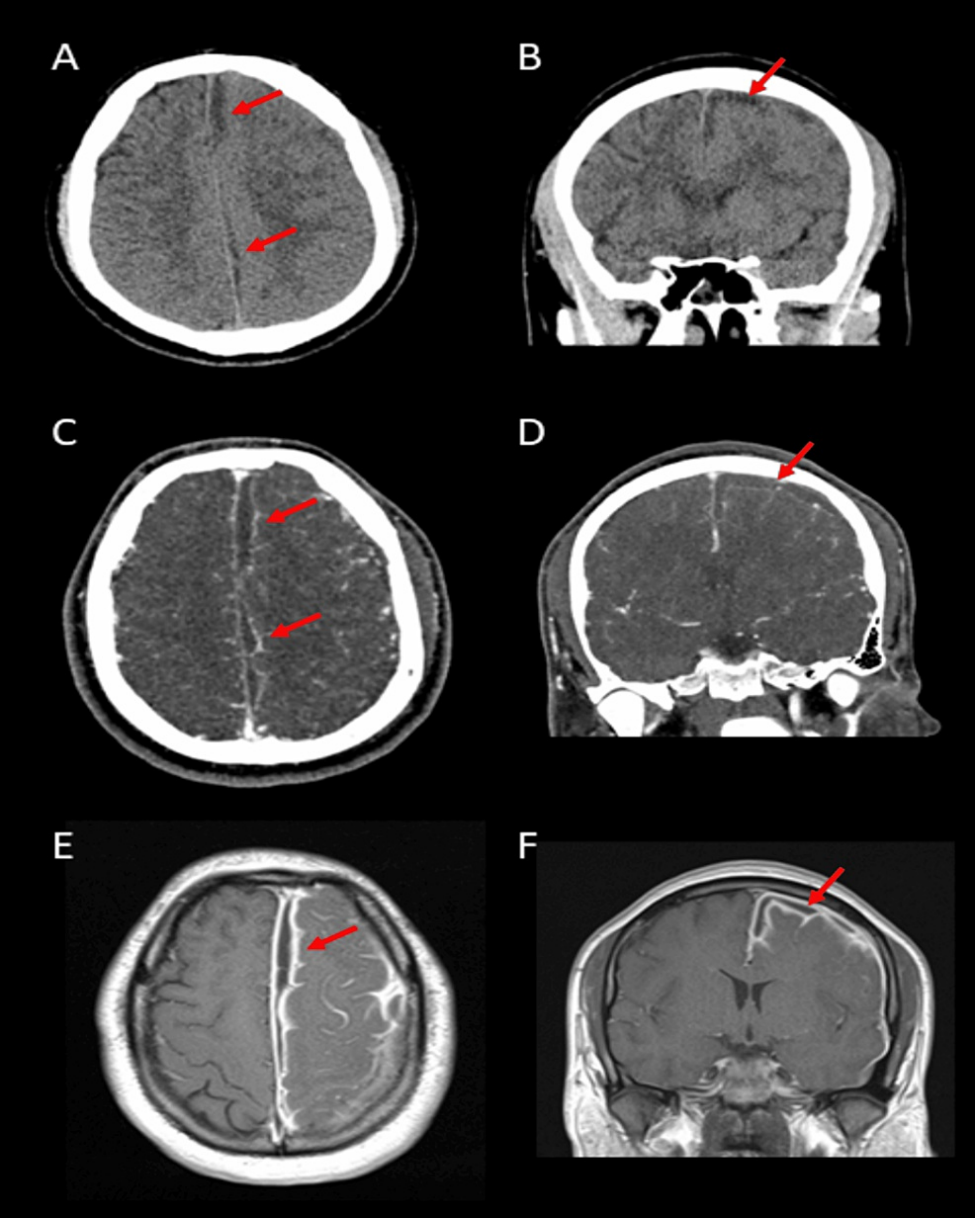
Imaging of the subdural empyema Head CT without contrast (A, B) demonstrates the subdural empyema (red arrows) along the falx cerebri and cerebral convexity on the left. Axial (C) and coronal (D) views of the head CT angiogram show the left subdural empyema (red arrows) with the absence of contrast extravasation. Post-contrast T1-weighted brain MRI (E, F) demonstrates diffuse enhancement of the meninges around the left subdural empyema (red arrows).

On day five of the patient’s hospital stay, he experienced sudden onset right-sided weakness. A clinical examination revealed right hemiplegia. A non-contrast CT of the head, a CT scan of the head and neck angiogram, and an MRI of the brain with and without gadolinium demonstrated an enhancing intracranial subdural empyema extending along the lining of the left falx cerebri and along the convexity of the left cerebrum (Figures 2A-2F).

An urgent neurosurgery consult was obtained, and a decompressive left craniectomy with evacuation and drainage was performed, demonstrating frank pus. The patient was then transferred to his room with a significant improvement in his weakness. Subsequent cultures from the subdural fluid collection did not grow any microorganisms, and the patient was treated empirically with antibiotics using cefepime, vancomycin, and metronidazole. The patient improved postoperatively from 0/5 to 4/5 in motor strength and was discharged to an acute rehabilitation facility with a six-week course of intravenous antibiotics. The patient later underwent synthetic cranioplasty after he was cleared for cranioplasty by an infectious disease consultant.

## Discussion

Intracranial subdural empyema is a loculated collection of pus in the subdural space. Subdural empyema is commonly caused by maxillofacial infections such as sinusitis, mastoiditis, and otitis, as well as traumatic skull fractures and post-surgical complications [1]. As the subdural space itself lacks anatomical barriers, subdural empyemas have the potential to extend across the surface of the brain, spanning the convexity of the cerebral hemispheres and occasionally reaching the contralateral hemisphere or the posterior fossa. Given the limited space within the cranial cavity, an intracranial subdural empyema can induce severe neurological symptoms and, in extreme cases, may be fatal due to direct pressure on the brain. Therefore, intracranial subdural empyema is a neurosurgical emergency [2]. Magnetic resonance imaging with intravenous gadolinium is the neuro-diagnostic imaging study of choice for visualizing subdural empyema, which presents as a crescent-shaped or elliptical lesion that displays decreased signal intensity on T1-weighted images and enhancement following the administration of contrast [1]. The early identification and prompt drainage of subdural empyema, along with an aggressive antibiotic regimen, are currently the mainstays of treatment.

Besides neuroimaging, conducting laboratory tests can provide additional support for the suspected diagnosis. An elevated white blood cell count, known as leukocytosis, may suggest an active infection process. However, in individuals with compromised or suppressed immune systems, there might not always be an elevation in white blood cells. Depending on the patient's immune response, bacterial infections could trigger a leftward shift in white blood cells along with an increase in neutrophils. Additional laboratory investigations, such as measuring serum inflammation markers such as erythrocyte sedimentation rate (ESR) and C-reactive protein (CRP), can offer further evidence since these markers are indicative of acute inflammation. While some clinicians might recommend blood cultures early in the disease course to guide antibiotic therapy, it's worth noting that in cases of subdural empyema, blood cultures seldom identify the causative organism due to the encapsulated nature of the infection. Performing a spinal puncture is not recommended and can pose risks, particularly in patients with elevated intracranial pressure, potentially leading to a mass effect [3, 4].

The pathogenesis of intracranial subdural empyema due to orbital cellulitis includes two major mechanisms: direct seeding due to bone erosions or retrograde thrombophlebitis via the valveless diploe veins [3, 4]. Infections are often polymicrobial, with anaerobic Gram-positive cocci, *Streptococcus spp.*, *Staphylococcus spp.*, and anaerobic Gram-negative bacilli among the most commonly isolated organisms [4]. In 7%-53% of the cases, cultures do not yield the growth of any organism, such as our patient, possibly due to prior or concurrent antibiotic use or improper application of culture methods [4]. Thrombophlebitis in the context of subdural empyema can lead to venous stasis with thrombosis, infarction, and subsequent cerebral inflammation. In undrained subdural pus, the resultant edema and brain compression are major contributors to neurological deterioration [4]. Our case shows that orbital cellulitis may cause an intracranial subdural empyema that can present with sudden-onset neurological deficits warranting prompt neurosurgical intervention.

## Conclusions

Intracranial subdural empyema can be fatal. Subdural empyemas can present with sudden-onset neurological deficits warranting prompt neurosurgical intervention. Here, we present a case of a rare holohemispheric intracranial subdural empyema spanning the entire left cerebral hemiconvexity. Early identification and prompt drainage of a subdural empyema, along with an aggressive and specific antibiotic regimen, is currently the mainstay of treatment to prevent complications and an unfavorable prognosis. Management requires an interdisciplinary team of providers that includes neurosurgeons, infectious disease specialists, critical care intensivists, and neurologists.
